# Bi-directional association between allergic rhinitis and diabetes mellitus from the national representative data of South Korea

**DOI:** 10.1038/s41598-021-83787-9

**Published:** 2021-02-23

**Authors:** Tae Kyung Lee, Ye Jin Jeon, Sun Jae Jung

**Affiliations:** 1 Yonsei University College of Medicine, Seoul, Korea; 2 Department of Public Health, Yonsei University Graduate School, Seoul, Korea; 3 Department of Preventive Medicine, Yonsei University College of Medicine, Seoul, Korea; 4 Department of Epidemiology, Harvard T.H. Chan School of Public Health, Boston, MA USA

**Keywords:** Diabetes, Respiratory distress syndrome, Epidemiology, Endocrinology, Medical research

## Abstract

Allergic rhinitis (AR) and diabetes mellitus (DM) share a common cause in inflammation; however, potential relationships between them are not clear. Therefore, we aimed to explore the associations between AR and DM. In this cross-sectional study, data were extracted from the Korean National Health and Nutrition Examination Survey 2007–2018 and weighted based on sociodemographic characters. AR and DM were defined by diagnoses thereof. Since self-reporting was not perfect, in order to complement the validity, we additionally performed sensitivity analysis by defining DM according to HbA1c and medication history. After excluding invalid data, the number of final participants was 29,246 (mean age, 54.95 ± 14.27 years). We calculated the odds ratio (OR) of newly self-reported DM among AR patients without DM history by multivariable logistic regression adjusted for potential confounders. A reverse association was also assessed. Patients with AR showed lower ORs for DM than those without AR (OR, 95% CI: men, 0.28, 0.19–0.42; women, 0.33, 0.24–0.46). Similarly, DM patients showed lower ORs for AR than patients without DM (men, 0.41, 0.31–0.56; women, 0.34, 0.25–0.46). The same results were obtained in sensitivity analysis defining DM according to HbA1c levels or DM treatment and in stratification analysis for age, residency, comorbidity, BMI, and menopause. In conclusion, we discovered that AR and DM show mutual inverse associations, regardless of sex, in individuals aged 30 years and older.

## Introduction

Allergic rhinitis (AR) affects 16.7% of Korean adults according to Korean Health Statistics 2018^1^. AR, with symptoms of sneezing, rhinorrhea, and nasal congestion, has an immense effect on one’s life. Research has shown that AR is mediated by type 1 allergic reactions related to helper T cell-1 (Th 1)^2^. Since AR is related to inflammation, several studies have explored relationships between AR and other diseases related to immune reactions, including cancer^3^, cardiovascular disease^4^, stroke^5^, and depressive disorder^6^.

Type 2 diabetes mellitus (DM) should be monitored and controlled carefully because it could cause complications such as hyperosmolar hyperglycemic state, blindness, neuropathy, and chronic renal failure^7^. In fact, some studies on the pathophysiology of DM have shown that DM is induced by insulin resistance in association with low-grade chronic inflammation^8,9^. Since the pathophysiologies of both AR and DM are related to inflammation, it would be reasonable to infer potential correlations between AR and DM, similar to cancer, cardiovascular disease, stroke, and depressive disorder, and documenting an association between AR and DM could provide novel perspectives on both diseases.

Previous studies have explored the associations between AR and patients with DM and its related conditions, such as metabolic syndrome and high-fasting plasma glucose (FPG)^10,11^. Hashimoto et al. found that subjects with high levels of FPG had a low prevalence of rhinitis. Hwang et al*.* suggested that subjects with metabolic syndrome had lower odds of AR than those without. Both studies only considered the effects of DM and its related conditions on AR occurrence, and not vice versa. In addition, they only defined exposure as DM and its related conditions, and not the DM itself, and Hashimoto et al*.* only included male participants. Unlike previous studies, we explored the bi-directional association of AR with DM itself, not DM-like conditions.

## Results

Table 1 shows the baseline characteristics of the study participants according to AR and DM: the upper part is organized according to AR status and the lower according to DM. In total, 29,246 participants (12,466 men and 16,780 women) were included for analysis. There were significant differences between the non-AR and AR groups for both sexes in age, family income, education, employment, marital status, urban residency, smoking, drinking, sleep deprivation, and dyslipidemia. Differences in overweight or obese and in the presence of comorbidity were significant only in women and men, respectively. Similarly, there were significant differences between the non-DM and DM groups for both sexes in family income, education, employment, marital status, smoking, drinking, overweight or obese, and presence of comorbidity. Sleep deprivation, however, was not significant for either sex. The covariates that could lead to a significant difference in AR or DM occurrence were adjusted in all of the analyses in this study, as shown in Figs. 2 and 3.

**Table 1:** Baseline characteristics of the study participants, organized by allergic rhinitis (AR) and diabetes mellitus (DM) (N = 29,112; 29,125).

Organized by allergic rhinitis (AR)	Men (N = 12,466)					Women (N = 16,780)				
	No AR (N = 11,181)		AR (N = 1285)		*p*-value	No AR (N = 14,443)		AR (N = 2337)		*p*-value
	N	(%)	N	(%)		N	(%)	N	(%)	
Age at least 62 years (N, %)	4172	(23.70)	287	(13.04)	< .001	5417	(29.59)	396	(12.05)	< .001
High family income (N, %)*	4574	(45.80)	658	(53.59)	< .001	5483	(41.57)	1123	(49.73)	< .001
High education (N, %)^†^	7625	(75.14)	1066	(87.20)	< .001	7887	(60.72)	1765	(78.91)	< .001
Employed (N, %)^‡^	8205	(79.67)	1046	(87.09)	< .001	6896	(49.20)	1241	(54.12)	< .001
Married (N, %)^§^	9348	(82.01)	1055	(79.43)	0.006	10,495	(74.60)	1826	(79.67)	< .001
Urban residency (N, %)^||^	7513	(69.02)	932	(72.60)	0.025	9840	(69.36)	1694	(73.95)	< .001
Ever Smoking (N, %)	9007	(80.25)	951	(74.23)	< .001	1327	(9.99)	304	(13.60)	< .001
Frequent drinking (N, %)^¶^	1596	(13.59)	119	(9.05)	< .001	345	(2.50)	45	(2.16)	< .001
Overweight/obese (BMI ≥ 23.0) (N, %)	7419	(67.64)	862	(68.19)	0.923	7931	(52.86)	1121	(46.70)	< .001
Sleep deprivation (N, %)^#^	852	(8.07)	148	(11.18)	< .001	1849	(13.03)	427	(17.81)	< .001
Dyslipidemia (N, %)	1781	(14.26)	224	(15.37)	< .001	3148	(19.16)	393	(14.09)	< .001
Comorbidity (N, %)**	5713	(44.44)	692	(49.12)	0.005	8157	(52.12)	1338	(54.62)	0.051

Organized by diabetes mellitus (DM)	Men (N = 12,466)					Women (N = 16,780)				
	No DM (N = 10,902)		DM (N = 1564)		*p*-value	No DM (N = 15,116)		DM (N = 1664)		*p*-value
	N	(%)	N	(%)		N	(%)	N	(%)	
Age at least 62 years (N, %)	3474	(19.54)	985	(49.39)	< .001	4617	(23.34)	1196	(65.48)	< .001
High family income (N, %)*	4812	(48.30)	420	(32.04)	< .001	6308	(44.86)	298	(20.71)	< .001
High education (N, %)^†^	7639	(51.60)	498	(32.08)	< .001	7864	(78.58)	827	(57.69)	< .001
Employed (N, %)^‡^	8378	(82.50)	873	(62.38)	< .001	7639	(51.60)	498	(32.08)	< .001
Married (N, %)^§^	9065	(81.32)	1338	(85.51)	< .001	11,374	(77.05)	947	(57.29)	< .001
Urban residency (N, %)^||^	7423	(69.74)	1022	(66.53)	0.038	10,426	(70.31)	1108	(67.27)	0.056
Ever Smoking (N, %)	8678	(79.34)	1280	(81.64)	< .001	1489	(10.73)	142	(8.40)	< .001
Frequent drinking (N, %)^¶^	1503	(13.06)	212	(13.17)	< .001	361	(2.54)	29	(1.51)	< .001
Overweight/obese (BMI ≥ 23.0) (N, %)	7198	(67.45)	1083	(70.19)	0.030	7838	(49.93)	1214	(73.28)	< .001
Sleep deprivation (N, %)^#^	865	(8.36)	135	(8.94)	0.516	2040	(13.62)	236	(15.13)	0.057
Dyslipidemia (N, %)	1399	(11.48)	606	(40.76)	**N/A**	2660	(15.36)	881	(50.87)	**N/A**
Comorbidity (N, %)**	5151	(41.59)	1254	(75.88)	< .001	8041	(49.47)	1454	(84.87)	< .001

*DM* diabetes mellitus, *SD* standard deviation.

*Monthly family income is more than 4 million won.

^†^Above the high-school graduation.

^‡^Currently employed.

^§^Currently married and lived with their husband (or wife).

||Seoul, other metropolitan cities, and the district which grade is 'dong' in Gyeon-gi province.

^¶^Drink more than 4 times a week.

^#^Hard to fall in or retain sleep status at least once a week and also feel tired in the day, simultaneously.

**Get more than one disease, including liver cirrhosis, hepatitis B, C, chronic renal failure, macular degeneration, glaucoma, cataract, otitis media, sinusitis, atopic dermatitis, major depression, cancers (thyroid, lung, endocardial, breast, large intestine, liver, stomach, and others 1, 2), thyroid benign disease, asthma, pulmonary tuberculosis, arthritis, myocardial infarction or angina, stroke, and hypertension.

### Bi-directional association between AR and DM

**Figure 1:**
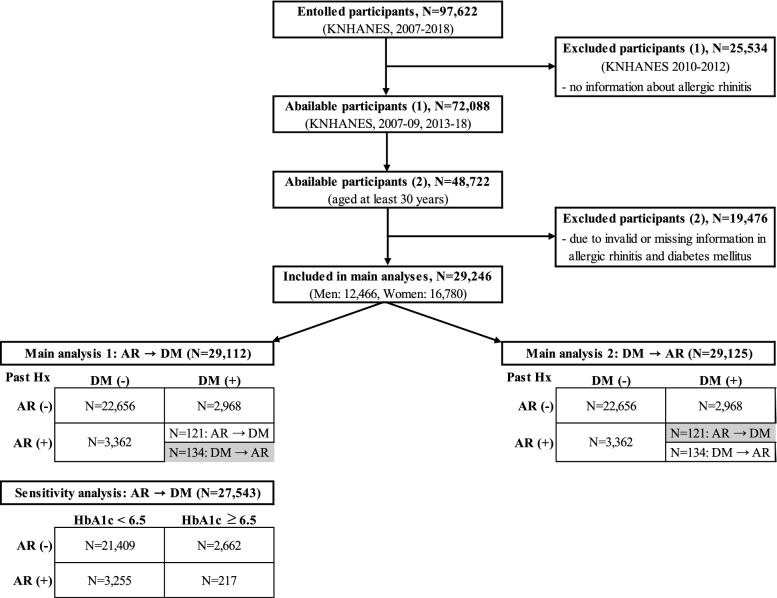
Flow chart of study participants. Sensitivity analysis, re-ran main analysis 1 by defining diabetes mellitus by HbA1c (cut-off: 6.5%). *KNHANES* Korea National Health and Nutrition Examination Survey, *AR* Allergic Rhinitis, *DM* Diabetes Mellitus, *Hx* history, *HbA1c* Hemoglobin A1c.

The upper part of the table shows the association between AR and DM based on the number of participants whose DM was not diagnosed before AR (main analysis 1 in Fig. 1). The total number of subjects in this part was 29,112 (12,397 men and 16,715 women). For men and women, the prevalence of DM was significantly lower in those with AR than those without AR, after adjusting for all confounders in this study (upper part of Table 2). Specifically, the ORs for DM were 0.28 and 0.33 in men and women, respectively. In addition, we also conducted sensitivity analysis defining DM according to an HbA1c level of > 6.5% or DM treatment history (Supplementary Table 3)^12^. Among the final study participants (N = 29,246), 1703 participants were excluded due to missing values of HbA1c. With this definition, likewise, the results showed that DM risk was also reduced in AR patients. The lower part of Table 2 shows the association of DM to AR. This table was based on data for participants whose AR had not been diagnosed before DM (main analysis 2 in Fig. 1). The total number of subjects was 29,125 (12,411 men and 16,714 women). The prevalence of AR in those with DM was significantly lower after adjusting for all covariates, regardless of sex. The ORs for AR were 0.41 and 0.34 in men and women, respectively.

**Table 2:** Bi-directional association between allergic rhinitis (AR) and diabetes mellitus (DM) (N = 29,112; 29,125).

	Group N	DM occurrence (%)	AR → DM (N = 29,112)	
			Fully adjusted model*	
			OR	(95% CI)
**Men (N = 12,397)**				
No AR	11,181	1437 (12.85)	1.00	(ref)
AR	1216	58 (4.77)	0.28	(0.19–0.42)
**Women (N = 16,715)**				
No AR	14,443	1531 (10.60)	1.00	(ref)
AR	2272	68 (2.99)	0.34	(0.24–0.47)

	Group N	AR occurrence (%)	DM → AR (N = 29,125)	
			Fully adjusted model*	
			OR	(95% CI)
**Men (N = 12,441)**				
No DM	10,902	1158 (10.62)	1.00	(ref)
DM	1509	72 (4.77)	0.41	(0.31–0.56)
**Women (N = 16,714)**				
No DM	15,116	2204 (14.58)	1.00	(ref)
DM	1598	67 (4.19)	0.34	(0.25–0.46)

*Adjusted for age, family income, education status, marital status, smoking, drinking frequency, sleep deprivation, dyslipidemia, BMI category (cut-off: 23.0 kg/m^2^), and the number of comorbidities.

*AR* allergic rhinitis, *DM* diabetes mellitus, *OR* odds ratio, *CI* confidence interval.

### Stratified analyses according to age, residency, comorbidity, BMI, and menopausal status

In stratified analysis, likewise un-stratified analysis of this study, we also adjusted all covariates including age, family income, education status, marital status, smoking, drinking frequency, sleep deprivation, dyslipidemia, BMI, and the number of comorbidities. Age was divided into three groups: 30–46, 47–61, and ≥ 62. As shown in Table 3, for the group with DM not diagnosed before AR, the OR and 95% CI were significantly low in all age groups. Both rural and urban showed significant OR and 95% CI. Similar with general results in Table 2, group stratified by the presence of comorbidities, overweight/obese (BMI ≥ 23.0 in Asian population^13^), and menopause also showed that significant outcomes. Thus, even in stratification analysis, DM occurrence in AR patients was lower than non-AR. Likewise, even in stratified groups, as shown in Supplementary Table 4, AR occurrence in DM patients was lower than non-DM.

**Table 3:** Stratified analysis for association of AR to DM regarding to age group, urban residency, comorbid, BMI, and menopause. (N = 29,112).

Subgroup (N of men/women)		AR → DM (N = 29,112)							
		Men (N = 12,397)				Women (N = 16,715)			
		No DM	DM	OR*	(95% CI)	No DM	DM	OR*	(95% CI)
**Age group**									
Aged 30–46 years (N = 4082/5475)	No AR (ref)	3365	97	0.23	(0.08–0.72)	4195	64	0.37	(0.16–0.86)
	AR	612	8			1203	13		
Aged 47–61 years (N = 3899/5464)	No AR (ref)	3128	419	0.37	(0.21–0.66)	4426	341	0.32	(0.19–0.53)
	AR	323	29			675	22		
Aged at least 62 years (N = 4416/5776)	No AR (ref)	3251	921	0.20	(0.11–0.37)	4291	1126	0.29	(0.19–0.45)
	AR	223	21			326	33		
**Residency**^**†**^									
Rural (N = 4009/5231)	No AR (ref)	3156	512	0.23	(0.10–0.53)	4082	521	0.41	(0.24–0.71)
	AR	323	18			608	20		
Urban (N = 8388/11,484)	No AR (ref)	6588	925	0.31	(0.19–0.49)	8830	1010	0.31	(0.21–0.46)
	AR	835	40			1596	48		
**Comorbidity**^**‡**^									
No comorbidity (N = 6050/7280)	No AR (ref)	5182	286	0.31	(0.13–0.72)	6088	198	0.26	(0.11–0.64)
	AR	569	13			987	7		
Comorbid (N = 6347/9435)	No AR (ref)	4562	1151	0.27	(0.17–0.42)	6824	1333	0.36	(0.25–0.51)
	AR	589	45			1217	61		
**BMI group**^**§**^									
Underweight/normal range (BMI < 23.0) (N = 3294/7670)	No AR (ref)	3294	444	0.15	(0.05–0.44)	6067	408	0.33	(0.18–0.59)
	AR	384	12			1176	19		
Overweight/obese (BMI ≥ 23.0) (N = 8236/8003)	No AR (ref)	6427	992	0.34	(0.22–0.52)	6813	118	0.33	(0.22–0.47)
	AR	771	46			1025	47		
**Menopausal status**^**||**^									
Pre-menopause (N = 0/8384)	No AR (ref)	N/A				6526	334	0.34	(0.20–0.55)
	AR					1505	19		
Menopause (N = 0/8163)	No AR (ref)					6284	1153	0.32	(0.22–0.45)
	AR					691	35		

*AR* allergic rhinitis, *DM* diabetes mellitus, *OR* odds ratio, *CI* confidence interval, *BMI* body mass index.

*Adjusted for age, family income, education status, marital status, smoking, drinking frequency, sleep deprivation, dyslipidemia, BMI category (cut-off: 23.0 kg/m^2^), and the number of comorbidities.

^†^Urban includes Seoul, other metropolitan cities, and the district which grade is ‘Dong’ in Gyeon-gi province. The other area is rural.

^‡^Comorbid is defined as status that get more one disease, including liver cirrhosis, hepatitis B, C, chronic renal failure, macular degeneration, glaucoma, cataract, otitis media, sinusitis, atopic dermatitis, major depression, cancers (thyroid, lung, endocervical, breast, large intestine, liver, stomach, and others 1, 2), thyroid benign disease, asthma, pulmonary tuberculosis, arthritis, myocardial infarction or angina, stroke, and hypertension.

^§^BMI classified according to Asian-Pacific criteria defined by World Health Organization.

||Menopause includes natural and artificial menopause. The other status is all pre-menopause.

## Discussion

Our results showed that the prevalence of DM is significantly lower in AR patients and that of AR is significantly lower in DM patients among both men and women. In other words, AR patients had a lower likelihood of DM occurrence than patients without AR. Likewise, DM patients had a lower likelihood of AR occurrence than those without DM. To confirm the robustness of our findings, we performed sensitivity analysis by defining DM according to HbA1c or diabetes treatment. Furthermore, the results of stratification analysis by age, residency, comorbidity, BMI, and menopause were highly comparable. Thus, it is reasonable to suggest that AR inhibits the occurrence of DM and that DM inhibits AR occurrence regardless of sex.

Several previous studies have explored the association between AR and DM-like conditions, such as metabolic syndrome, impaired fasting glucose, and high FPG^10,11^. In Hashimoto et al., involving 10,093 male participants, they calculated the ORs of rhinitis in five groups divided by FPG level. They found that subjects with high levels of FPG had a low prevalence of rhinitis. Hwang et al. suggested that among 30,590 subjects, the ORs of AR were lower in participants with metabolic syndrome or impaired fasting glucose than in the healthy population. They, however, only focused on the effect of DM-like conditions on AR, not actual DM. High FPG does not mean diabetes, because plasma glucose could be increased by many other factors and may also be masked by therapeutic drugs for diabetes. Metabolic syndrome, likewise, is not same as diabetes. In other words, both studies only considered the effects of DM-like conditions on AR occurrence and not vice versa. Thus, we used actual DM as a variable and elucidated the bidirectional inhibitory associations between AR and DM.

Although this study was cross-sectional in its design, we considered the temporality of the diagnosis, considering the Bradford-Hill criteria^14^, when evaluating the associations between AR and DM. Since causal relationships could not be confirmed in this study due to cross-sectional design, we considered the sequence of diagnoses of AR and DM to enhance the validity of the association. Furthermore, strict exclusion criteria, consideration of many confounders, and stratification analysis also contributed to the validity.

Plausibility is also an important factor for confirmation of a causal relationship based on the Bradford-Hill criteria^14^. In other words, when a result can be explained by biologically well-known mechanisms, their association would be much more rigorous than a simple association. Our results might possibly be explained by the pathophysiology of helper T (Th) cell polarization, where Th1 and Th2 cells counteract each other. When immunologic stimulation is generated, premature Th cells would be differentiated into Th1, 2, and 17 cells^15^. Since the number of premature T cells is limited, domination of one type of Th cell suppresses the activation of the other type of Th cells by cytokine reactions. This phenomenon is called Th polarization^16^. In light of this theory, it is well known that AR is mediated by Th 2 domination^2^, and according to a recent review by Kartika et al., type 2 DM is induced by Th 1 domination^8^. With Th polarization, AR and type 2 DM downregulate reciprocally. Thus, our conclusion could satisfy the plausibility of the Bradford-Hill criteria, which reinforced the validity of the association we presented.

As mentioned above, the strengths of this study were the novelty of the topic and thorough evaluation of the associations, considering temporality. In addition, the phenomenon in the study might possibly be explained biologically. Thus, we consider the association between AR and DM to be meaningful.

There are several limitations in this study. First of all, this was a cross-sectional study that could not reveal causation and could represent only association^17^. To address this issue, we considered temporal sequence of AR and DM diagnoses in the analysis. However, consideration of temporality could not fully ensure the causality, for example, we could utilize the age by year unit, which may not distinguish the overlap perfectly. Additionally, the measurement of the age of diagnosis of each disease may not be as same as the age of biological onset. Therefore, subjects, as an example, whose AR diagnosis was slightly prior to DM, might have been the patient who originally had DM prior to AR. To investigate this issue, we conducted a sensitivity analysis, excluding the gap under 1 year between both diseases (Supplementary Table 2), and the result was similar with the original outcomes.

Second, the main exposure and outcome of this study were defined by self-reported questionnaires, which could lead to measurement bias, including recall bias based on a recall. Participants also could respond incorrectly, especially in the case of AR, which could lead to underestimation. In contrast, the agreement between self-reported and measured data for diabetes was relatively high (k = 0.82) in a previous validation study of KNHANES^18^. We also conducted the sensitivity analysis, comparing self-reported and clinically identified DM, showed the similar results (Supplementary Table 3). However, in the sensitivity analysis, 1703 participants were excluded due to missing value of HbA1c, who showed partially different general characteristics compared to included participants (Supplementary Table 5). Specifically, excluded participants were more likely to have low social economic status, be unmarried or obese, and have more comorbidities than included participants (result not shown). Therefore, it was possible that our results might have overestimated the true association regarding AR and DM.

Thirdly, since KNHANES did not discriminate detail DM types, so we could not use data of pure type 2 diabetes (T2D). To reduce heterogeneity of DM data and make more homogenous toward T2D, we restricted participant’s age over 30, but it was not exact T2D. Further studies with well discriminated data of DM types would be needed to discover exact association between T2D and AR.

To our knowledge, this study is the first to demonstrate that AR and DM have mutual inverse association. Since we considered the temporality of the diagnoses and the plausibility of the phenomenon might be explained by Th polarization, the association between AR and DM appears to be significant. This clinical phenomenon could be used as the basis for additional research on AR and DM. For example, in further studies, it might be possible to show that Th polarization modulation can be exploited to treat DM and AR. In fact, immunotherapy based on Th polarization is already being executed for AR treatment^19^. In the same manner, application of Th polarization in DM treatment could also be possible. With this study as the starting point, we can expect novel effective regimens of treatment for DM to be developed in the future.

In conclusion, we found that AR and DM have mutual inverse associations in both men and women aged over 30 years. Th polarization could be one of a possible explanation for these findings. Our results could provide a novel perspective on the mutuality between AR and DM and open new pathways to additional research on AR and DM.

## Materials and methods

### Participants

Data was extracted from a cross-sectional study, Korean National Health and Nutrition Examination Survey (KNHANES) 2007–2018 which was a nationwide population-based self-questionnaire survey conducted by the Korean Ministry of Health and Welfare. Participants were recruited by a two-stage stratified cluster sampling. Sampling frame was the latest population and housing census, conducted by Statistics Korea and supplemented by the data of public housing price conducted by Ministry of Land, Infrastructure and Transport, Korea. Stratification variables were administrative district, sex age, type of housing, and area of living space. Trained interviewers performed the interviews using structured questionnaires to obtain information including sociodemographic factors, health-related factors, lifestyle factors, the use of medical services and female reproductive factors. Data on lifestyle were recorded by self-reported questionnaire. HbA1c levels were measured via high performance liquid chromatography (HLC-723G7; Tosoh, Tokyo, Japan). All data of the participants was weighted based on a sociodemographic character and significance because KNHANES data represented total population of Korea. To be more specific, the Rao-scott chi‐square test for complex samples was used to compare the differences in the exposure group (PROC SURVEYFREQ) and the complex sample multiple logistic regression (PROC SURVEYLOGISTIC) was used to determine the bi-directional association between AR and DM.

Among KNHANES 2007-2018, total number of study participants are 97,622. To determine the association between AR and DM, we excluded the data of 68,376 participants. First of all, from the 2010–2012 data, 25,534 participants were excluded due to absence of assessment of AR. Secondly, among participants aged under 30 years, 23,366 subjects were excluded to increase the homogeneity of the DM group, as near to type 2 DM. Lastly, patients with missing value on AR and DM were excluded (17,527 and 1949 participants, respectively). Subsequently, 29,246 participants (12,466 men and 16,780 women, respectively), aged over 30 years, were included (Fig. 1).

### Definitions of allergic rhinitis and diabetes mellitus

In the KNHANES study, AR and DM were reported via an interview questionnaire. AR and DM patients were identified using self-reported history of a clinical diagnosis of AR and DM. Cases of “DM incidence after AR diagnosis” were defined according to age at DM and AR diagnosis. Cases of “AR incidence after DM diagnosis” were also defined in the same way. For sensitivity analysis, individuals with HbA1c levels above 6.5% (48 mmol/mol) or with current DM treatment was defined as DM patients. (Glycated hemoglobin (HbA1c) is a form of hemoglobin bonded to a sugar and the target analyze of a blood test for diagnosing and monitoring DM.) The reason why we conducted the main analysis by defining DM through self-reporting, rather than HbA1c, was based on our main goal to evaluate the temporal sequence between DM and AR. In this study, to clarify the temporal sequence of each association between AR → DM and DM → AR, we utilized the data regarding the age of diagnosis for both DM and AR. HbA1c was not proper for comparing temporality, since it only reflected the state at the latest measurement. Therefore, we chose self-reported morbidities as the main exposure and outcome measurements. For the AR → DM association evaluation, we additionally conducted sensitivity analysis by defining DM based on HbA1c and medication history.

### Covariates

Since the goal of this study was to assess the risk of DM in AR patients and vice versa, we considered several confounders known to affect both AR and DM occurrence, such as age, family income, education, marital status, ever smoking status, drinking frequency, BMI, sleep deprivation, dyslipidemia, the number of comorbidities, residence, and menopause. “Family income” was defined by monthly earnings, and it was divided into four groups: income under 150, 150–316, 316–518, and over 518 Korean-won (unit). “Education level” was assigned to seven categories, including pre-school, graduating from elementary school, middle school, high school, college, university, graduate school. “Marital status” was defined by four categories as follows: married and living with spouse, married but not living together or divorced, bereaved, and unmarried. “Ever smoking” was divided into three categories, including never, quit now, and still smoking. “Drinking frequency” was assigned to three categories, including less than once per month, two to four times per month, and more than two times per week. BMI (kg/m^2^) was divided into two categories, underweight/normal (under 23.0 kg/m^2^) and overweight/obese (above 23.0 kg/m^2^), according to the WHO-WPRO (Western Pacific Regional Office) guideline; normal range was 18.5 to 23^20^. Overweight and underweight were over 23.5 and under 18.5 respectively. Our database included a small population of underweight group, which could lack statistical power due to the small sample; therefore, we did not divide the under-23 group into two categories. We defined sleep disturbance as a few days’ experience of waking from sleep and difficulty going to sleep with lethargy in the daytime. “Sleep deprivation” was based on the weekly frequency of sleep disturbance: not at all, a few days, more than a week, and every day. The “number of comorbidities” was the sum of comorbidities assessed in the KNHANES, including liver cirrhosis, hepatitis C, hepatitis B, chronic renal failure, macular degeneration, glaucoma, cataract, otitis media, sinusitis, atopic dermatitis, depressive disorder, cancer (thyroid, lung, cervix, breast, colon, liver, gastric, and others), benign thyroid disease, asthma, tuberculosis, arthritis, myocardial ischemia or angina, stroke, and hypertension. Lastly, “residence” was divided into urban and rural: urban areas included Seoul, other metropolitan cities, and areas whose administrative district was larger than “dong” in Gyeonggi province; rural included all non-urban areas. All covariates were coded as categorical variables. In case of missing values in these variables, we coded them as a separate category; this allowed us to include the information on exposure and outcome variables from the entire individual in the major statistical analysis without omitting the individuals with any missing values present in the covariates. (e.g. missing/unknown was coded as “9”; family income, n = 104; education, n = 189; marital status, n = 24; smoking status, n = 249, drinking frequency, n = 4027, dyslipidemia, n = 73; BMI category, n = 71; sleep deprivation, n = 14,514).

### Statistical analysis

To assess the risk of DM in AR patients and vice versa, we excluded some participants group from all of them for each case. To be more specific, by using the value of ‘age of diagnosis’ in database, we compared diagnosis order between AR and DM for each participant. All participants were divided into 5 types. Type A was the subject who had both diseases and AR was diagnosed earlier than DM. Type B was the subject who had both, and DM was diagnosed earlier than AR. Type C was the people only diagnosed AR and type D was only DM. Type E was the people who was not diagnosed both AR and DM. There were two main analyses in this study (Fig. 1). Firstly, in main analysis 1, to evaluate the effects of AR on DM occurrence, we used all participants except type B group. Likewise, in main analysis 2, we used all participants except type A, to evaluate the effects of DM on AR. By considering temporality, we could evaluate the association between AR and DM. Since men and women have different physiologies, we analyzed the data of men and women separately.

**Figure 2:**
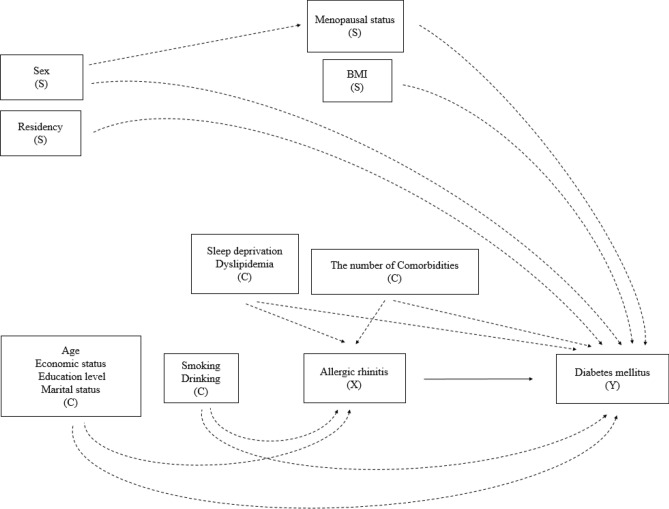
Assumed structure of the association regarding the effects of allergic rhinitis (AR; exposure X) with covariates (C) on diabetes mellitus (DM; outcome Y) with stratification (S).

**Figure 3:**
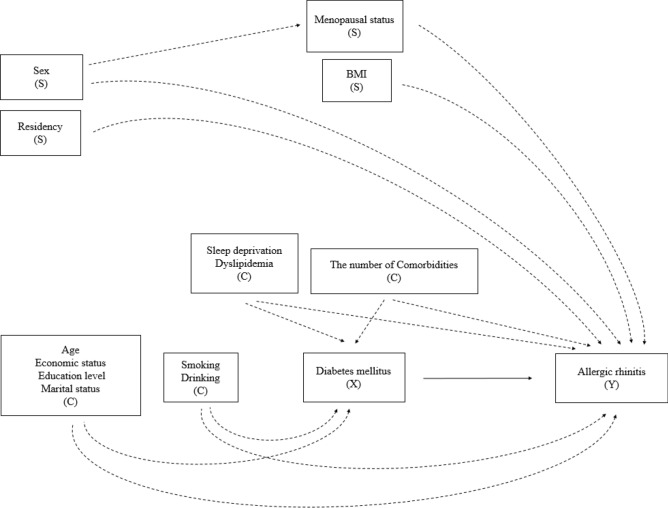
Assumed structure of the association regarding the effects of diabetes mellitus (DM; exposure X) with covariates (C) on allergic rhinitis (AR; outcome Y) with stratification (S).

Multivariable logistic regression models were used to determine the associations between AR and DM (Figs. 2 and 3). We calculated the odds ratios (ORs) and 95% CI (confidence intervals) of DM prevalence in subjects with AR. In the counter direction, we also calculated the ORs and 95% CI of AR prevalence in subjects with DM. In each direction of analysis, we added the following covariates to the final model: age, family income, education, marital status, ever smoking status, drinking frequency, BMI (cut-off: 23.0 kg/m^2^), sleep deprivation, dyslipidemia, and the number of comorbidities. Stratified analysis was also performed by age group, residence, presence of comorbidity, BMI group, and menopause (-in women only). All analyses were performed using the statistical software package SAS version 9.4 (SAS Institute Inc., Cary, NC, USA), and weight distribution of the population was considered. Since both AR and DM had sex difference in many aspects of the diseases^21,22^, to evaluate precisely, all analyzing process had done in each sex, respectively.

## Supplementary Information

Supplementary Information

## Data Availability

The Korea Centers for Disease Control and Prevention (KCDC) has supported researchers in Korea by providing annual workshops for data users. The KCDC has published the Korea Health Statistics each year, and microdata are publicly available through the KNHANES website (http://knhanes.cdc.go.kr).
