# Supplementary material for: Bi-directional association between allergic rhinitis and diabetes mellitus from the national representative data of South Korea
Source: Sci Rep. 2021 Feb 23;11:4344. doi: 10.1038/s41598-021-83787-9 (PMC7902822; doi:10.1038/s41598-021-83787-9)
Supplement: Supplementary file 1 — Supplementary Information [file 41598_2021_83787_MOESM1_ESM.docx]

**Title: Bi-directional association between Allergic Rhinitis and Diabetes Mellitus from the national representative data of South Korea**

**Authors:** Tae Kyung Lee^1^, Ye Jin Jeon, MPH^2^, Sun Jae Jung, MD, PhD^3,4^

^1^ Yonsei University College of Medicine, Seoul, Korea

^2^ Department of Public Health, Yonsei University Graduate School, Seoul, Korea

^3^ Department of Preventive Medicine, Yonsei University College of Medicine, Seoul, Korea

^4^ Department of Epidemiology, Harvard T.H. Chan School of Public Health, Boston, MA, USA

**Supplementary Tables**

**Supplementary Table 1.** Bi-directional association between allergic rhinitis (AR) and diabetes mellitus (DM) in model 1 to 3 (N=29,112; 29,125)

**Supplementary Table 2.** Bi-directional association between allergic rhinitis (AR) and diabetes mellitus (DM) with 1year lag-time (N=29,090; 29,095)

**Supplementary Table 3.** Sensitivity analysis of allergic rhinitis (AR) effects on diabetes mellitus (DM) with different criteria. (N=27,543)

**Supplementary Table 4.** Stratified analysis for association of diabetes mellitus (DM) to allergic rhinitis (AR) regarding to age group, urban residency, comorbid, and menopause. (N=29,125)

**Supplementary Table 5.** Comparison between included (N=27,543) and excluded (N=1,703) participants in sensitivity analysis due to missing HbA1c

**Supplementary Table 1. Bi-directional association between allergic rhinitis (AR) and diabetes mellitus (DM) in model 1 to 3 (N=29,112; 29,125)**

|  | Group N | DM occurrence (%) | **AR→DM (N=29,112)** | | | | | | | |
| --- | --- | --- | --- | --- | --- | --- | --- | --- | --- | --- |
|  |  |  | Model 1^*^ | |  | Model 2^†^ | |  | Model 3^‡^ | |
|  |  |  | OR | (95% CI) |  | OR | (95% CI) |  | OR | (95% CI) |
| Men (N=12,397) | |  |  |  |  |  |  |  |  |  |
| No AR | 11,181 | 1,437 (12.85) | 1.00 | (ref) |  | 1.00 | (ref) |  | 1.00 | (ref) |
| AR | 1,216 | 58 (4.77) | 0.44 | (0.32 - 0.60) |  | 0.46 | (0.33 - 0.63) |  | 0.46 | (0.33 - 0.64) |
| Women (N=16,715) | |  |  |  |  |  |  |  |  |  |
| No AR | 14,443 | 1,531 (10.60) | 1.00 | (ref) |  | 1.00 | (ref) |  | 1.00 | (ref) |
| AR | 2,272 | 68 (2.99) | 0.44 | (0.33 - 0.59) |  | 0.46 | (0.34 - 0.61) |  | 0.46 | (0.34 - 0.60) |
|  | Group N | AR occurrence (%) | **DM→AR (N=29,125)** | | | | | | | |
|  |  |  | Model 1^*^ | |  | Model 2^†^ | |  | Model 3^‡^ | |
|  |  |  | OR | (95% CI) |  | OR | (95% CI) |  | OR | (95% CI) |
| Men (N=12,441) | |  |  |  |  |  |  |  |  |  |
| No DM | 10,902 | 1,158 (10.62) | 1.00 | (ref) |  | 1.00 | (ref) |  | 1.00 | (ref) |
| DM | 1,509 | 72 (4.77) | 0.55 | (0.41 - 0.73) |  | 0.57 | (0.43 - 0.76) |  | 0.58 | (0.43 - 0.77) |
| Women (N=16,714) | |  |  |  |  |  |  |  |  |  |
| No DM | 15,116 | 2,204 (14.58) | 1.00 | (ref) |  | 1.00 | (ref) |  | 1.00 | (ref) |
| DM | 1,598 | 67 (4.19) | 0.43 | (0.33 - 0.58) |  | 0.45 | (0.34 - 0.59) |  | 0.45 | (0.34 - 0.60) |

*Adjusted for age

†Adjusted for age, family income, education status, and marital status

‡Adjusted for age, family income, education status, marital status, smoking, and drinking frequency

**Abbreviations**: AR, allergic rhinitis; DM, diabetes mellitus; OR, odds ratio; CI, confidence interval

**Supplementary Table 2. Bi-directional association between allergic rhinitis (AR) and diabetes mellitus (DM) with 1year lag-time (N=29,090; 29,095)**

|  | Group N | DM occurrence (%) | **AR→DM (N=29,090)** | | | | | | | | | | |
| --- | --- | --- | --- | --- | --- | --- | --- | --- | --- | --- | --- | --- | --- |
|  |  |  | Model 1^*^ | |  | Model 2^†^ | |  | Model 3^‡^ | |  | Model 4^§^ | |
|  |  |  | OR | (95% CI) |  | OR | (95% CI) |  | OR | (95% CI) |  | OR | (95% CI) |
| Men (N=12,389) | |  |  |  |  |  |  |  |  |  |  |  |  |
| No AR | 11,181 | 1427 (12.85) | 1.00 | (ref) |  | 1.00 | (ref) |  | 1.00 | (ref) |  | 1.00 | (ref) |
| AR | 1,208 | 50 (4.14) | 0.39 | (0.27 - 0.54) |  | 0.40 | (0.28 - 0.56) |  | 0.40 | (0.29 - 0.57) |  | 0.24 | (0.16 - 0.37) |
| Women (N=16,711) | |  |  |  |  |  |  |  |  |  |  |  |  |
| No AR | 14,443 | 1531 (10.60) | 1.00 | (ref) |  | 1.00 | (ref) |  | 1.00 | (ref) |  | 1.00 | (ref) |
| AR | 2,268 | 64 (2.82) | 0.42 | (0.31 - 0.56) |  | 0.43 | (0.32 - 0.58) |  | 0.43 | (0.32 - 0.58) |  | 0.32 | (0.23 - 0.44) |
|  | Group N | AR occurrence (%) | **DM→AR (N=29,095)** | | | | | | | | | | |
|  |  |  | Model 1^*^ | |  | Model 2^†^ | |  | Model 3^‡^ | |  | Model 4^§^ | |
|  |  |  | OR | (95% CI) |  | OR | (95% CI) |  | OR | (95% CI) |  | OR | (95% CI) |
| Men (N=12,396) | |  |  |  |  |  |  |  |  |  |  |  |  |
| No DM | 10,902 | 1,158 (10.62) | 1.00 | (ref) |  | 1.00 | (ref) |  | 1.00 | (ref) |  | 1.00 | (ref) |
| DM | 1,494 | 57 (3.82) | 0.42 | (0.30 - 0.57) |  | 0.42 | (0.30 - 0.57) |  | 0.42 | (0.30 - 0.58) |  | 0.34 | (0.25 - 0.46) |
| Women (N=16,699) | |  |  |  |  |  |  |  |  |  |  |  |  |
| No DM | 15,116 | 2,204 (14.58) | 1.00 | (ref) |  | 1.00 | (ref) |  | 1.00 | (ref) |  | 1.00 | (ref) |
| DM | 1,583 | 52 (3.28) | 0.35 | (0.26 - 0.48) |  | 0.35 | (0.26 - 0.48) |  | 0.35 | (0.26 - 0.48) |  | 0.27 | (0.19 - 0.38) |

* Adjusted for age.

† Adjusted for age, family income, education status and marital status.

‡ Adjusted for age, family income, education status, marital status, smoking and drinking frequency.

§ Adjusted for age, family income, education status, marital status, smoking, drinking frequency, sleep deprivation, dyslipidemia, BMI category (cut-off: 23.0 kg/m^2^), and the number of comorbidities.

**Abbreviations**: AR, allergic rhinitis; DM, diabetes mellitus; OR, odds ratio; CI, confidence interval

**Supplementary Table 3. Sensitivity analysis of allergic rhinitis (AR) effects on diabetes mellitus (DM) with different criteria. (N=27,543)**

|  | Group N | DM occurrence (%) | Diabetes Mellitus (HbA1c ≥ 6.5% or treated) (N=27,543) | | | | | | | | | | |
| --- | --- | --- | --- | --- | --- | --- | --- | --- | --- | --- | --- | --- | --- |
|  |  |  | Model 1^*^ | |  | Model 2^†^ | |  | Model 3^‡^ | |  | Model 4^§^ | |
|  |  |  | OR | (95% CI) |  | OR | (95% CI) |  | OR | (95% CI) |  | OR | (95% CI) |
| Men (N=11,920) | |  |  |  |  |  |  |  |  |  |  |  |  |
| No AR | 10,675 | 1,339 (12.54) | 1.00 | (ref) |  | 1.00 | (ref) |  | 1.00 | (ref) |  | 1.00 | (ref) |
| AR | 1,245 | 99 (7.95) | 0.82 | (0.66 - 1.02) |  | 0.85 | (0.68 - 1.05) |  | 0.86 | (0.69 - 1.08) |  | 0.64 | (0.50 - 0.82) |
| Women (N=15,623) | |  |  |  |  |  |  |  |  |  |  |  |  |
| No AR | 13,396 | 1,323 (9.88) | 1.00 | (ref) |  | 1.00 | (ref) |  | 1.00 | (ref) |  | 1.00 | (ref) |
| AR | 2,227 | 118 (5.30) | 0.82 | (0.67 - 0.998) |  | 0.84 | (0.69 - 1.04) |  | 0.84 | (0.69 - 1.03) |  | 0.73 | (0.61 - 0.88) |

* Adjusted for age.

† Adjusted for age, family income, education status and marital status.

‡ Adjusted for age, family income, education status, marital status, smoking and drinking frequency.

§ Adjusted for age, family income, education status, marital status, smoking, drinking frequency, sleep deprivation, dyslipidemia, BMI category (cut-off: 23.0 kg/m^2^), and the number of comorbidities.

**Abbreviations**: AR, allergic rhinitis; DM, diabetes mellitus; OR, odds ratio; CI, confidence interval

**Supplementary Table 4. Stratified analysis for association of diabetes mellitus (DM) to allergic rhinitis (AR) regarding to age group, urban residency, comorbid, and menopause. (N=29,125)**

| subgroup (N of men / women) | | **DM→AR (N=29,125)** | | | | | | | | | |
| --- | --- | --- | --- | --- | --- | --- | --- | --- | --- | --- | --- |
|  |  | Men (N=12,441) | | | | |  | Women (N=16,714) | | | |
|  |  | No AR | AR | OR^*^ | (95% CI) | |  | No AR | AR | OR^*^ | (95% CI) |
| **Age group** |  |  |  |  |  | |  |  |  |  |  |
| aged 30-46 years  (N=4,081 / 5,475) | No DM (ref) | 3,365 | 612 | 0.21 | (0.06 - 0.71) | |  | 4,195 | 1,203 | 0.44 | (0.22 - 0.87) |
|  | DM | 97 | 7 |  |  |  |  | 64 | 13 |  |  |
| aged 47-61 years  (N=3,890 / 5,457) | No DM (ref) | 3,128 | 323 | 0.28 | (0.16 - 0.50) | |  | 4,426 | 645 | 0.19 | (0.11 - 0.35) |
|  | DM | 419 | 20 |  |  |  |  | 341 | 15 |  |  |
| aged at least 62 years  (N=4,440 / 5,782) | No DM (ref) | 3,251 | 223 | 0.59 | (0.41 - 0.83) | |  | 4,291 | 326 | 0.42 | (0.28 - 0.64) |
|  | DM | 921 | 45 |  |  |  |  | 1,126 | 39 |  |  |
| **Residency^†^** |  |  |  |  |  | |  |  |  |  |  |
| rural (N=4,005 / 5,226) | No DM (ref) | 3,156 | 323 | 0.30 | (0.14 - 0.66) | |  | 4,082 | 608 | 0.19 | (0.10 - 0.35) |
|  | DM | 512 | 14 |  |  |  |  | 521 | 15 |  |  |
| urban (N=8,406 / 11,488) | No DM (ref) | 6,588 | 835 | 0.43 | (0.30 - 0.60) | |  | 8,830 | 1,596 | 0.39 | (0.28 - 0.54) |
|  | DM | 925 | 58 |  |  |  |  | 1010 | 52 |  |  |
| **Comorbidity^‡^** |  |  |  |  |  | |  |  |  |  |  |
| no comorbidity  (N=6,048 / 7,278) | No DM (ref) | 5,182 | 569 | 0.27 | (0.11 - 0.67) | |  | 6,088 | 987 | 0.11 | (0.04 - 0.36) |
|  | DM | 286 | 11 |  |  |  |  | 198 | 5 |  |  |
| comorbid  (N=6,363 / 9,436) | No DM (ref) | 4,562 | 589 | 0.41 | (0.29 - 0.59) | |  | 6,824 | 1,217 | 0.39 | (0.29 - 0.53) |
|  | DM | 1,151 | 61 |  |  |  |  | 1,333 | 62 |  |  |
| **BMI group^§^** |  |  |  |  |  | |  |  |  |  |  |
| underweight/normal range  (BMI < 23.0) (N=4,146 / 7,665) | No DM (ref) | 3294 | 384 | 0.32 | (0.18 - 0.56) | |  | 6,067 | 1176 | 0.25 | (0.13 - 0.47) |
|  | AR | 444 | 24 |  |  |  |  | 408 | 14 |  |  |
| overweight/obese  (BMI ≥ 23.0) (N=8,238 / 9,007) | No DM (ref) | 6427 | 771 | 0.44 | (0.30 - 0.64) | |  | 6,813 | 1025 | 0.37 | (0.26 - 0.52) |
|  | AR | 992 | 48 |  |  |  |  | 1,118 | 51 |  |  |
| **Menopausal status^\|\|^** |  |  |  |  | |  |  |  |  |  |  |
| pre-menopause (N= 0 / 8,384) | No DM (ref) | N/A | | | | |  | 6,526 | 1,505 | 0.35 | (0.21 - 0.56) |
|  | DM |  |  |  |  |  |  | 334 | 19 |  |  |
| menopause (N= 0 / 8,161) | No DM (ref) |  |  |  |  |  |  | 6,284 | 691 | 0.29 | (0.21 - 0.42) |
|  | DM |  |  |  |  |  |  | 1,153 | 33 |  |  |

* Adjusted for age, family income, education status, marital status, smoking, drinking frequency, sleep deprivation, dyslipidemia, BMI category (cut-off: 23.0 kg/m2), and the number of comorbidities.

† Urban includes Seoul, other metropolitan cities, and the district which grade is 'Dong' in Gyeon-gi province. The other area is rural.

‡ Comorbid is defined as status that get more one disease, including liver cirrhosis, hepatitis B, C, chronic renal failure, macular degeneration, glaucoma, cataract, otitis media, sinusitis, atopic dermatitis, major depression, cancers (thyroid, lung, endocervical, breast, large intestine, liver, stomach, and others 1, 2), thyroid benign disease, asthma, pulmonary tuberculosis, arthritis, myocardial infarction or angina, stroke, and hypertension.

§ BMI classified according to Asian-Pacific criteria defined by World Health Organization

|| Menopause includes natural and artificial menopause. The other status is all pre-menopause.

**Abbreviations**: AR, allergic rhinitis; DM, diabetes mellitus; OR, odds ratio; CI, confidence interval; BMI, body mass index

**Supplementary Table 5. Comparison between included (N=27,543) and excluded (N=1,703) participants in sensitivity analysis due to missing HbA1c.**

|  | Missing value in HbA1c | | | |
| --- | --- | --- | --- | --- |
|  | No (N=27,543) | | Yes (N=1,703) | |
|  | N | (%) | N | (%) |
| Gender, male (N, %) | 11,920 | (49.06) | 546 | (34.95) |
| Age at least 62 years (N, %) | 9,253 | (23.47) | 1019 | (48.60) |
| High family income (N, %)^*^ | 11,428 | (43.14) | 410 | (29.27) |
| High education (N, %)^†^ | 17,661 | (70.94) | 682 | (48.67) |
| Employed (N, %)^‡^ | 16,769 | (65.99) | 619 | (41.69) |
| Married (N, %)^§^ | 21,614 | (79.09) | 1,110 | (66.53) |
| Urban residency (N, %)^\|\|^ | 18,925 | (70.10) | 1,054 | (63.46) |
| Ever Smoking (N, %) | 11,022 | (44.30) | 567 | (36.43) |
| Frequent drinking (N, %)^¶^ | 1,986 | (7.60) | 119 | (7.40) |
| Overweight/obese (BMI ≥ 23.0) (N, %) | 16,411 | (59.86) | 922 | (54.07) |
| Sleep deprivation (N, %)^#^ | 3,075 | (11.14) | 201 | (11.76) |
| Dyslipidemia (N, %) | 5,205 | (16.40) | 341 | (17.48) |
| Comorbidity (N, %)^**^ | 14,717 | (48.08) | 1,183 | (62.89) |

*Monthly family income of more than 4 million Korean won

†Above high-school graduation

‡Currently employed

§Currently married and living with spouse

||Seoul, other metropolitan cities, and the district with a grade of “dong” in Gyeon-gi province.

¶Drinks more than 4 times a week

#Hard to fall asleep or retain sleep status at least once a week, and also feels tired in the day at the same time

**Has more than one disease, including liver cirrhosis, hepatitis B or C, chronic renal failure, macular degeneration, glaucoma, cataract, otitis media, sinusitis, atopic dermatitis, major depression, cancers (thyroid, lung, endocardial, breast, large intestine, liver, stomach, and others 1, 2), thyroid benign disease, asthma, pulmonary tuberculosis, arthritis, myocardial infarction or angina, stroke, and hypertension

**Abbreviations**: DM, diabetes mellitus; SD, standard deviation.
